# Influência da Composição Racial Brasileira no Controle da Pressão Arterial: A Necessidade de Novos Olhares além do Tratamento Medicamentoso

**DOI:** 10.36660/abc.20220063

**Published:** 2022-03-10

**Authors:** Weimar Kunz Sebba Barroso, Sandro Rodrigues Batista, Priscila Valverde de Oliveira Vitorino, Ana Luiza Lima Sousa

**Affiliations:** 1 Liga de Hipertensão Arterial Universidade Federal de Goiás Goiânia GO Brasil Liga de Hipertensão Arterial, Universidade Federal de Goiás, Goiânia, GO – Brasil; 2 Departamento de Clínica Médica Faculdade de Medicina Universidade Federal de Goiás Goiânia GO Brasil Departamento de Clínica Médica, Faculdade de Medicina, Universidade Federal de Goiás, Goiânia, GO – Brasil; 3 Secretaria de Estado da Saúde de Goiás Goiânia GO Brasil Secretaria de Estado da Saúde de Goiás, Goiânia, GO – Brasil; 4 Pontifícia Universidade Católica de Goiás Escola de Ciências Sociais e da Saúde Goiânia GO Brasil Pontifícia Universidade Católica de Goiás – Escola de Ciências Sociais e da Saúde,Goiânia, GO – Brasil; 5 Faculdade de Enfermagem Universidade Federal de Goiás Goiânia GO Brasil Faculdade de Enfermagem, Universidade Federal de Goiás, Goiânia, GO – Brasil

**Keywords:** Hipertensão, Grupos Étnicos, Doença Crônica

**“ *Of all the forms of inequality, injustice in health care is the***

***most shocking and inhumane* ”**

*Martin Luter King, 1966*

No contexto da saúde cardiovascular, algumas características raciais são frequentemente associadas a um pior controle da pressão arterial (PA); adultos negros, por exemplo, apresentam hipertensão arterial mais grave e resistente quando comparados a outros grupos étnicos.^1 - 3^ Quase todas essas evidências foram geradas em contextos populacionais com pouca miscigenação e, por isso, é imperativo o entendimento do impacto das características raciais específicas da população brasileira sobre ocorrência, diagnóstico e controle da hipertensão arterial.^4 , 5^

O estudo apresentado por Sousa et al.6 traz um novo olhar em relação à questão racial e a sua influência no tratamento e controle da PA em adultos brasileiros. Usando um robusto banco de dados do Estudo Longitudinal da Saúde do Adulto (ELSA-Brasil), avaliaram a associação entre raça/cor da pele autorrelatadas e controle de PA em indivíduos utilizando várias classes de anti-hipertensivos em monoterapia, publicação complementar do grupo à influência da etnia sobre aspectos da doença hipertensiva.^7 , 8^

Indivíduos negros em uso de inibidores da enzima conversora de angiotensina (IECA), bloqueadores de receptor de angiotensina (BRA), diuréticos tiazídicos (DT) e betabloqueadores (BB) em monoterapia apresentaram pior controle de PA em relação aos brancos. Após tratamento estatístico adequado, os autores concluíram que as diferenças de controle de PA entre os vários grupos raciais não podem ser explicadas por uma possível menor eficácia dos IECA e BRA em indivíduos negros. Embora observacional, o estudo começa a desvendar alguns tópicos importantes no manejo de pacientes com hipertensão arterial, pertencentes a alguns grupos específicos (neste caso, a população negra), além de vislumbrar algumas hipóteses que precisam ser investigadas, sobretudo aquelas relacionadas às iniquidades em saúde dessa população e a suas repercussões.

A miscigenação racial, característica da população brasileira, traz grandes questões socioeconômicas ao país e, de forma associada, desafios importantes para o cuidado em saúde.^4 , 5^ Na perspectiva dos determinantes sociais do processo saúde doença, fatores relacionados à composição racial de uma população (incluindo racismo) podem contribuir para a ocorrência de iniquidades em saúde e, assim, influenciar negativamente os desfechos relacionados.^1 , 2 , 9 , 10^ Desta forma, dificuldades no acesso aos serviços de saúde, às medidas de prevenção e proteção da saúde e aos tratamentos mais adequados podem ser frequentes. De acordo com dados da Pesquisa Nacional por Amostra de Domicílios (PNAD) (2019), 9,4% dos brasileiros se autorrelataram como pretos, e 46,8% como pardos.^11^

Ainda, precisamos citar características que contribuem sobremaneira para o manejo de doenças crônicas no Sistema Único de Saúde:^12^ o acelerado envelhecimento populacional e as crescentes desigualdades sociais associados ao acúmulo significativo de morbidades crônicas em nossa população (aproximadamente 26 milhões de brasileiros com ≥50 anos relatam mais de duas condições crônicas, e hipertensão arterial está presente na maioria das combinações entre essas).^13^ Cita-se também o aumento de incidência de todas as doenças crônicas não transmissíveis analisadas pela Pesquisa Nacional de Saúde (PNS) na comparação entre 2013 e 2019.^4^

Dados estratificados conforme raça/etnia da PNS 2019 mostram que brasileiros pretos ou pardos relataram pior estado de saúde, principalmente os do sexo feminino (57,8%). Além disso, realização de consulta médica nos últimos 12 meses foi mais frequente entre a população branca que na população negra, independente de sexo.^4^ Comparando-se as edições da PNS de 2013 e 2019, observou-se maior proporção de negros hipertensos que relataram uso de medicamentos, realização de exames complementares e consultas com especialistas em 2019. Esses pacientes também apresentaram maior taxa de assistência médica principalmente no serviço público e em Unidades Básicas de Saúde, embora a última consulta não tenha sido realizada com o mesmo médico das consultas anteriores.^5^ Ainda neste cenário, estudo brasileiro^7^ mostra que morar em bairros economicamente segregados, onde negros e pardos foram mais propensos a viver, está associado com maiores chances de hipertensão arterial (26%) e diabetes (50%) em relação a morar em outros bairros, podendo, assim, constituir um ambiente potencial de impulsão de desigualdades raciais relacionadas à ocorrência de fatores de risco cardiometabólicos.^7^

Assim, de forma complementar ao estudo de Sousa et al.,^6^ a sociedade brasileira demanda urgentemente por estudos que tragam as várias dimensões do cuidado, desde o acesso aos serviços de saúde, medicamentos, exames diagnósticos e especialistas, até o cuidado longitudinal e coordenado por equipe multidisciplinar e estratégias de autocuidado associadas às intersecções de suas características socioeconômicas, demográficas e culturais.
