# Supplementary material for: Influência da Composição Racial Brasileira no Controle da Pressão Arterial: A Necessidade de Novos Olhares além do Tratamento Medicamentoso
Source: Arq Bras Cardiol. 2022 Mar 10;118(3):623–4. [Article in Portuguese] doi: 10.36660/abc.20220063 (PMC8959038; doi:10.36660/abc.20220063)
Supplement: Translation

# Translation: Influence of Racial Composition on Blood Pressure Control in the Brazilian Population: The Need for New Perspectives Beyond Drug Treatment

**Authors:** Weimar Kunz Sebba Barroso, Sandro Rodrigues Batista, Priscila Valverde de Oliveira Vitorino, Ana Luiza Lima Sousa

**Affiliations:** 1 Brazil Liga de Hipertensão Arterial, Universidade Federal de Goiás, Goiânia, GO – Brazil; 2 Brazil Departamento de Clínica Médica, Faculdade de Medicina, Universidade Federal de Goiás, Goiânia, GO -– Brazil; 3 Brazil Secretaria de Estado da Saúde de Goiás, Goiânia, GO – Brazil; 4 Brazil Pontifícia Universidade Católica de Goiás – Escola de Ciências Sociais e da Saúde, Goiânia, GO – Brazil; 5 Brazil Faculdade de Enfermagem, Universidade Federal de Goiás, Goiânia, GO – Brazil

**Keywords:** Hypertension, Ethnic Groups, Chronic Disease

**“ *Of all the forms of inequality, injustice in health care is the***

***most shocking and inhumane* ”**

*Martin Luter King, 1966*

In the context of cardiovascular health, some racial characteristics have been frequently associated with worse blood pressure (BP) control. For example, Black adults have more severe resistant hypertension as compared with other ethnic groups.^1 - 3^ A lot of this evidence has been gathered from populations where there had been little racial mixing and, for this reason, understanding the impact of specific racial characteristics of the Brazilian population on the occurrence, diagnosis, and control of hypertension is imperative.^4 , 5^

The study by Sousa et al.^6^ provides a new perspective on the influence of race on the treatment and control of BP in Brazilian adults. Using a robust database of the Brazilian Longitudinal Study of Adult Health (ELSA-Brasil), the authors evaluated the association of self-reported race/skin color with BP control in individuals under different monotherapy antihypertensive regimens. This publication complements previous studies of the group on the influence of ethnicity on several aspects of hypertensive disease.^7 , 8^

Black individuals using angiotensin-converting enzyme inhibitors (ACEI), angiotensin receptor blockers (ARBs), thiazide diuretics (TD) and beta blockers showed a worse BP control than White individuals. After statistical treatment of the data, the authors concluded that the differences in BP control between the racial groups could not be explained by a possible lower efficacy of ACEI and ARBs in Black patients. Despite the observational nature of the study, it starts to reveal some important topics about the management of hypertensive patients belonging to specific groups (in this case, Black population), and put forward some hypotheses that need to be investigated, mainly those related to racial health inequality and its repercussions.

Racial mixing, characteristic of the Brazilian population, raises important socioeconomical questions, and important challenges to health care in the country.^4 , 5^ From the perspective of social determinants of health and disease processes, factors related to racial composition of the population (including racism) can contribute to health inequality, and thereby negatively influence the outcomes.^1 , 2 , 9 , 10^ Difficulty in accessing health services, preventive and protective measures, and adequate treatment may be common. According to the 2019 Brazilian National Household Sample Survey (PNAD, Pesquisa Nacional por Amostra de Domicílios), 9.4% of respondents self-reported as Black, and 46.8% as *Pardo* .^11^

Also, we need to mention some features that strongly contribute to the management of chronic diseases in the Brazilian Unified Health System:^12^ the accelerated population aging and increasing social inequality, associated with the marked increase of chronic morbidities in our population (approximately 26 million people aged ≥ 50 years report more than two chronic conditions, where hypertension is present in most of them).^13^ There is also the increase in the incidence of all non-communicable diseases from 2013 to 2019, as reported in the Brazilian National Health Survey (PNS).^4^

In fact, data stratified by race/ethnicity from the 2019 PNS showed that Black and *pardo* individuals, mainly women (57.8%), reported worse health status. Besides, White people reported higher attendance at medical appointments than Black people, regardless of sex.^4^ As compared with the 2013 PNS, a higher proportion of Black hypertensive patients who self-reported use of medications, performance of complementary tests and visits to specialists was found in the 2019 PNS. These patients also showed higher rates of attendance at medical care, especially in public services and primary health centers, although they were seen by different physicians at the last visit from the ones in previous consultations.^5^ Also, a Brazilian study showed that living in economically segregated neighborhoods, where Black and Pardo people are more likely to live, is associated with higher odds of hypertension (26%) and diabetes (50%) as compared with living in other areas. Thus, economically segregated neighborhoods may represent potential environment for promoting racial inequalities regarding the occurrence of cardiometabolic risk factors.^7^

Therefore, extending the findings of Sousa et al.,^6^ the Brazilian population urgently calls for further studies that provide a wider perspective of care, from access to health services, medications, diagnostic tests, and specialists, to a longitudinal, coordinated multidisciplinary care and self-care strategies consistent with their socioeconomic, demographic and cultural characteristics.
